# Citronellol Reduces Sepsis-Induced Renal Inflammation via AP-1/NF-κB/TNF-α Pathway

**DOI:** 10.3390/biom15111614

**Published:** 2025-11-17

**Authors:** Huda Rashid Atiyah, Sarmed H. Kathem, Surya M. Nauli

**Affiliations:** 1 Pharmacology and Toxicology Department, College of Pharmacy, University of Kufa, Kufa 66001, Iraq; 2 Pharmacology and Toxicology Department, College of Pharmacy, University of Baghdad, Baghdad 10046, Iraq; skathem@copharm.uobaghdad.edu.iq; 3 Department of Biomedical & Pharmaceutical Sciences, School of Pharmacy, Chapman University, Irvine, CA 992866, USA; nauli@chapman.edu

**Keywords:** TNF-α, NF-κB, AP-1, KIM-1, CLP, kidney injury, citronellol

## Abstract

Sepsis is characterized by the over-production of pro-inflammatory cytokines. Cecal ligation and puncture (CLP) is a well-accepted model for recreating sepsis-induced renal injury in mice. The current study investigates how citronellol, a naturally occurring substance with a variety of biological characteristics, can prevent acute kidney inflammation brought on by CLP. In the CLP mouse model, citronellol was administered orally at doses of 50 and 100 mg/kg. Serum levels of creatinine and urea were used as markers of renal function, and the Murine Sepsis Score (MSS) was used to assess the severity of sepsis. According to our findings, CLP caused a decline in renal function, as shown by higher serum urea and creatinine levels in comparison to control mice. Nevertheless, administering citronellol as pretreatment at doses of 50 and 100 mg/kg alleviated the deterioration in renal functions. Citronellol decreased levels of serum urea and creatinine. Citronellol demonstrated an anti-inflammatory effect by reducing pro-inflammatory cytokines (TNF-α, NF-κB, AP-1) and KIM-1. Overall, our study suggests that citronellol holds a promise as a potential therapeutic agent for mitigating kidney inflammation.

## 1. Introduction

Acute kidney injury (AKI) is a complex syndrome that results from heterogeneous mechanisms and carries considerable morbidity and mortality [1]. AKI occurs in 10–15% of hospitalized patients and 50% in intensive care unit (ICU) settings [2]. Sepsis is a devastating inflammatory response syndrome in clinical practice, and toxins, pathogenic bacteria, and their metabolic products entering the bloodstream are the main causes of sepsis [3]. Sepsis is a hyperinflammatory disease that causes a range of tissue damage and organ dysfunctions, including heart, kidney, and lung problems [4]. Sepsis is characterized by the excessive release of pro-inflammatory cytokines, including interleukin (IL)-1β, IL-6, IL-8, and tumor necrosis factor-α (TNF-α), which trigger the immune responses of the host leading to cytokine flair, hemodynamic instability, and ultimately organ dysfunction and septic shock. During sepsis, the stimulation of the sympathetic nervous system, the damage of endothelia, and the release of vasoactive substances, such as endothelin, vasopressin, and angiotensin II, lead to altered blood flow distribution and microcirculatory dysfunction [5]. These factors could damage the kidney tissue and trigger AKI by complex molecular mechanisms [6].

The cecal ligation and puncture (CLP) technique is a widely utilized method for creating sepsis model. Since the cecum contains a large number of bacteria, puncturing it leads to polymicrobial peritonitis. This, in turn, leads to the movement of bacteria into the blood (bacteremia), septic shock, multiple organ failure, and eventually death [7]. It is generally recognized that CLP provides a more accurate reflection of clinical reality than earlier methods, such as endotoxin injection. Thus, CLP is considered the gold standard for experimental induction and the investigation of the pathogenesis of sepsis [8,9].

Citronellol is a naturally occurring monoterpene alcohol (3, 7-Dimethyl-6-often-1-ol) found in several aromatic plant species, such as *Cymbopogon citratus*, *Cssymbopogon winterianus* and *Lippia alba*, commonly used as a fragrance and flavoring agent. Other industries that use citronellol include the food, cosmetics, and pharmaceutical sectors. There are two types of citronellol. The first type is (+)-CT (the dextrorotatory enantiomer of citronellol), which is mostly found in citronella, Amyris, and eucalyptus citriodora oils. The second type is the (-)-CT (the levorotatory enantiomer of citronellol), which is mostly found in pelargonium and rose oils (both are colorless liquids with a rose scent) [10,11]. Citronellol has also been demonstrated to possess antibacterial, antifungal, and repellent qualities in vitro, as well as cardiovascular protective, antidiabetic, and antinociceptive qualities in vivo [12,13].

The present study hypothesized that citronellol had a protective effect on the kidney function and pathology in a mouse model of cecal ligation and puncture-induced AKI. These studies uncovered unique processes underlie cecal ligation and puncture-induced AKI.

## 2. Materials and Methods

### 2.1. Reagents

Citronellol (CAS number: 106-22-9) was purchased from Sigma (New York, NY, USA). Formaldehyde was provided from Sinopharm chemical reagent Co., Ltd. (Shanghai, China). Serum urea and creatinine assay kit reagents were acquired from Linear chemical (Barcelona, Spain). KIM-1, NF-κB, AP-1, TNF-α, and GAPDH Primers were purchased from Macrogen (Seoul, Republic of Korea). RNA extraction kit (Catalog number: ER101-01) was purchased from TransGen biotech (Beijing, China). EasyScript^®^ One-Step gDNA Removal and cDNA Synthesis SuperMix (catalog number: AE311-03) were bought from TransGen biotech (China). TransStart^®^ Top Green qPCR Super Mix (catalog number: AQ601-01) was purchased from TransGen biotech (China).

### 2.2. Animal Experiments

The University of Baghdad’s College of Pharmacy supplied the mice utilized in this research; a total of 24 BALB/c albino male mice, each weighing an average of (24.3 ± 0.5 g), were involved. The mice were given unrestricted access to water and a standard diet. All animal procedures conducted complied with both institutional and international regulations concerning the ethical treatment and use of laboratory animals (approval number: 1917). To induce CLP, mice were anesthetized with 100 mg/kg ketamine (Alfasan, Woerden, Holland, catalog number: 36408/3000) and 10 mg/kg xylazine (Alfasan, Holland, Catalog number: 36408/3007) under anesthesia. Mice were positioned on their backs, and a 3 cm longitudinal incision was created in the lower abdomen after the skin was shaved and disinfected. The cecum was gently pulled out. A 3–0 suture was then used to securely ligate the cecum at its base, just below the ileocecal junction of the mouse’s intestine. A 19-gauge needle was utilized to create a single puncture in the cecum, allowing a small quantity of stool to be released through gentle pressure. The cecum was returned to the abdominal cavity, and the abdominal wall was sutured with a 3–0 suture. The skin was sutured shut afterward. Immediately after surgery, 1 mL of prewarmed normal saline was given subcutaneously; all animals were kept under the same conditions after the surgery [14]. In the negative control group, the cecum was located, but it was neither ligated nor punctured. All mice had free access to food and water after recovery from anesthesia.

**Figure 1:**
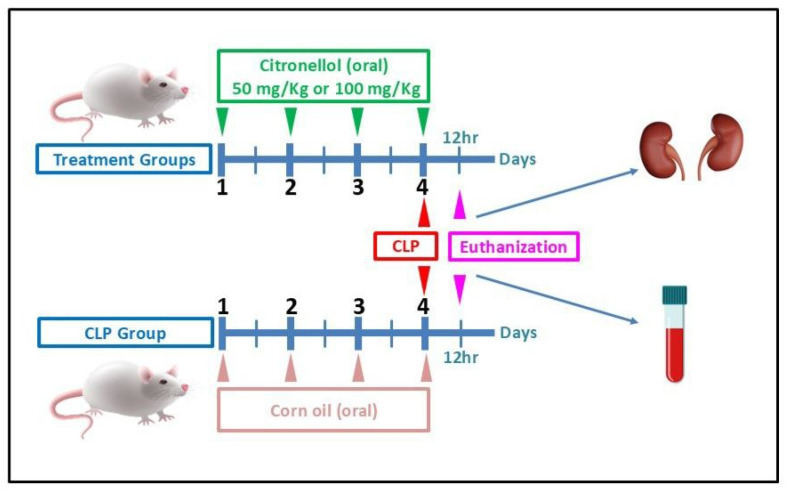
Schematic presentation of experimental animal design. CLP: cecal ligation and puncture; hr: hour.

Four groups of mice were used as follows (*n* = 6 for each group): Group I (Control): mice were orally given 0.1 mL corn oil for 4 days; cecum of these animals was located, but it was neither ligated nor punctured. Group II (the CLP model group): mice underwent CLP procedure as described above. Group III (50 mg/kg citronellol): mice were treated with 50 mg/kg citronellol orally for 4 days, and CLP was performed on day 4. Group IV (100 mg/kg citronellol): mice were treated with 100 mg/kg citronellol orally for 4 days, and CLP was performed on day 4. Euthanasia was conducted on day 4, precisely 12 h following CLP [14]; subsequently, blood and tissue specimens were gathered for examination. The scoring of AKI was performed by using the Jablonski score [15]. The experimental design is depicted in Figure 1.

### 2.3. Blood Collection

On the fourth day, blood was drawn using retro-orbital sampling. Subsequently, the blood sample was then left to coagulate for half an hour at room temperature. The samples were centrifuged at 4 °C for 20 min at 3000 rpm after coagulation. A micropipette was used to carefully extract the resultant serum, which was then transferred into micro-centrifuge tubes and labeled. These tubes were then frozen and stored to determine the urea and creatinine levels, which was performed within 24 h of collection, as described previously [16,17].

### 2.4. Biochemical Measurements

Creatinine (Cr) and serum urea levels were examined as key markers of severity of renal injury. The measurements were performed using a semi-automated biochemical in accordance with the manufacturer’s instructions. **Creatinine measurementwas based on an adaptation of the original picrate reaction (Jaffe) 1, in which creatinine combines with picrate ions in an alkaline environment to generate a crimson complex. Urea measurement was performed in a ready-made kit that utilized a colorimetric method. The principle of the assay involves hydrolyzing urea and urease producing ammonia and carbon dioxide.**

### 2.5. The Murine Sepsis Score (MSS) System

The MSS system used to assess the severity of sepsis was based on observational parameters. The MSS was scored based on the combinational factors of animal appearance (ruffled, hunched, and rigid), level of consciousness (activity, response to sound and touch), respiratory (rate, effort, or difficulty of breathing), eye appearance (open, closed, signs of discharge), and ability to right itself after being moved [18,19]. The scale of the score was ranged between 0 and 4. The final MSS is calculated by averaging the scores of each of those components for each group. This scoring system provides a valuable tool to assess the well-being of mice and to provide a measure of the general condition of mice with high specificity and sensitivity for predicting the onset of severe sepsis and death during the experimental timeline. The MSS was determined by an observer who remained unaware of the animal group or the treatment administered to ensure a rigorous assessment. The reported observations were carried out 12 h after CLP and immediately prior to euthanasia. Furthermore, the survival rate was computed for every group to offer an additional means of evaluating the severity of condition and contrasting it with the efficacy of treatment. In addition, the survival rate was calculated for all groups to provide a further tool to assess the severity of condition and compare it to the treatment effectiveness [20].

### 2.6. Jablonski (AKI) Score System Criteria

The cortical necrosis or necrosis of the renal cortex was graded on a scale of 0.5 to 4 according to the residual cortical change in a single longitudinal section. Specifically, we examined vacuolization or cortical damage (necrosis) according to the Jablonski scores for the assessment of renal damage [15,21,22]. A score of 0.5 was assigned for a small amount of focal damage on the cortical area; a score of 1 was given if about 10% of the cortical showed residual change; a score of 2 was given for 10–20% cortical necrosis; a score of 3 was given for 20–30% cortical necrosis; and a score of 4 was given for more than 30% necrosis. The histology of the injuries on the kidney samples of the animals in the 4 groups was evaluated according to the aforementioned score system by a blinded pathologist using standard light microscopy.

### 2.7. Gene Expression Analysis

All animals were euthanized with ketamine and xylazine, after which cervical dislocation was carried out 12 h after CLP [23]. The right kidney was removed and homogenized [24]. The expressions of NF-kB, AP-1, KIM-1, and TNF-α genes (mRNA) were quantified using qRT-PCR (quantitative reverse transcription–polymerase chain reaction), with GAPDH serving as the housekeeping gene [25]. Total RNA was extracted from the kidney homogenate using an RNA kit; subsequently, cDNA synthesis was performed using the EasyScript^®^ one-step gDNA (genomic DNA) removal and cDNA synthesis method. The mRNA expression levels were determined with SYBR Green Supermix. The primer sequences are provided in Table 1 and were designed using IDT’s PrimerQuest program.

### 2.8. Statistical Analysis

All statistical analyses were carried out with one-way ANOVA test followed by Tukey’s post hoc test for multiple groups. To identify significant differences between all groups, *p* < 0.05 was used as the threshold for statistical significance. Survival rate was analyzed using the Kaplan–Meier method. Data analyses were performed with the GraphPad Prism 8 software (Dotmatics).

## 3. Results

### 3.1. Effects of Citronellol on Inflammatory Pathways in CLP-Induced Kidney Injury

**Figure 2:**
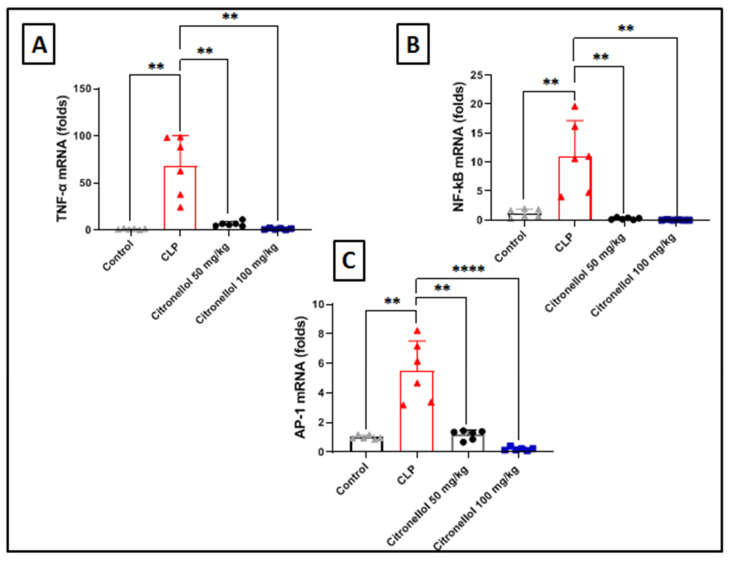
Effects of citronellol on inflammatory pathways in CLP-induced kidney injury in mice. Data represent mean ± SEM for mRNA expression of inflammatory markers measured 12 h after induction with CLP in mouse kidney tissue: (**A**) TNF-α; (**B**) NF-kB; (**C**) AP-1. Citronellol treatment used two different doses (50 mg/kg vs. 100 mg/kg) for four consecutive days before CLP. Calculations of gene expression were performed relative to GAPDH expression as a control gene in fold changes. *n* = 6 in each group. ** *p* < 0.01; **** *p* < 0.0001 indicate statistical significance. Control: 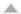; CLP: 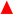; citronellol 50 mg/kg: 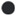; citronellol 100 mg/kg: 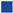.

The evaluation of inflammatory markers provided insight into the molecular players involved in kidney inflammation brought on by CLP. One of the key indicators of inflammation is TNF-α, and it was measured in this study. CLP in mice resulted in a significant spike in TNF-α (Figure 2A). The mRNA levels in comparison to the corresponding levels of the control mice (Figure 2A): 68.37 ± 13.07 vs. 1.16 ± 0.25 folds. Furthermore, mice pretreated with citronellol (both 50 mg/kg and 100 mg/kg) showed a significant decline in TNF-α gene expression (6.49 ± 1.04 and 1.25 ± 0.32 vs. 68.37 ± 13.07), in contrast to the mice that were not given any treatment. This finding suggests that in kidney inflammation brought on by CLP, citronellol had a potent anti-inflammatory effect. Transcription factors that are crucial for inflammatory signaling were evaluated; NF-κB (Figure 2B) and AP-1 (Figure 2C) were examined to achieve a better understanding of the pathways that citronellol targets. In this signaling study, citronellol administration (50 or 100 mg/kg) resulted in a remarkable decline in gene expressions of NF-κB (Figure 2B) compared to non-treated mice (0.26 ± 0.07 and 0.04 ± 0.02 vs. 11.04 ± 2.51). In the same context, mice treated with citronellol (50 and 100 mg/kg) showed significant attenuation in kidney AP-1 expression (Figure 2C) compared to that of non-treated mice (1.167 ± 0.13 and 0.197 ± 0.05 vs. 5.46 ± 0.84).

### 3.2. Effects of Citronellol on Murine Sepsis Score (MSS) and Survival Rate in CLP-Induced Kidney Injury

**Figure 3:**
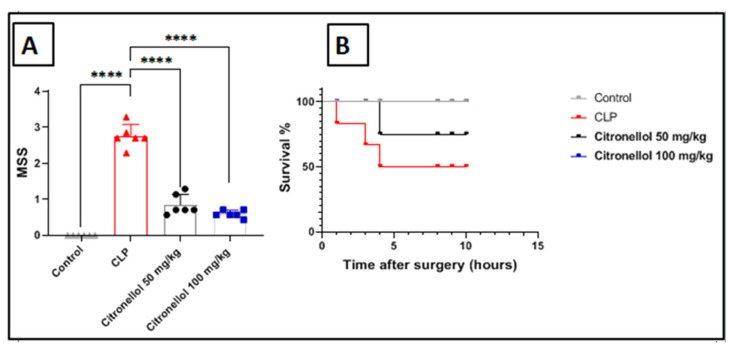
Effects of citronellol on Murine Sepsis Score (MSS) and survival rate in CLP-induced kidney injury in mouse. Data represented as mean ± SEM for: (**A**) MSS; (**B**) survival rate. Citronellol treatment used two different doses (50 mg/kg or 100 mg/kg) for four consecutive days before CLP. *n* = 6 in each group. **** *p* < 0.0001 indicate statistical significance. Control: 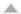; CLP: 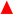; citronellol 50 mg/kg: 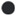; citronellol 100 mg/kg: 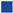.

In this research, the MSS was used to evaluate the severity of sepsis observed in each group. Figure 3A demonstrates a notable increase in MSS in the CLP group when compared to the control group (2.57 ± 0.2 vs. 0.02 ± 0.02), whereas a significant decrease in MSS was noted in groups treated with citronellol at doses of 50 mg/kg and 100 mg/kg compared to the CLP group (0.86 ± 0.16 and 0.64 ± 0.17 vs. 2.76 ± 0.24). These results suggest that citronellol contributes to a reduction in the severity of sepsis in mice. In addition to MSS, we also calculated the survival rate in this study. As shown in Figure 3B, a marked decline in the survival rate (50%) was observed in mice subjected to CLP after 12 h of the procedure, while a significant increase in survival rate (75%) was noted in the group receiving citronellol at 50 mg/kg. Notably, no fatalities were recorded in the group treated with 100 mg/kg of citronellol (survival rate 100%). These findings strongly support the conclusions from the measurements of other parameters in our study, indicating that citronellol may have the ability to alleviate the severity of sepsis in mice.

### 3.3. Effect of Citronellol on Urea and Creatinine in CLP-Induced Kidney Injury

**Figure 4:**
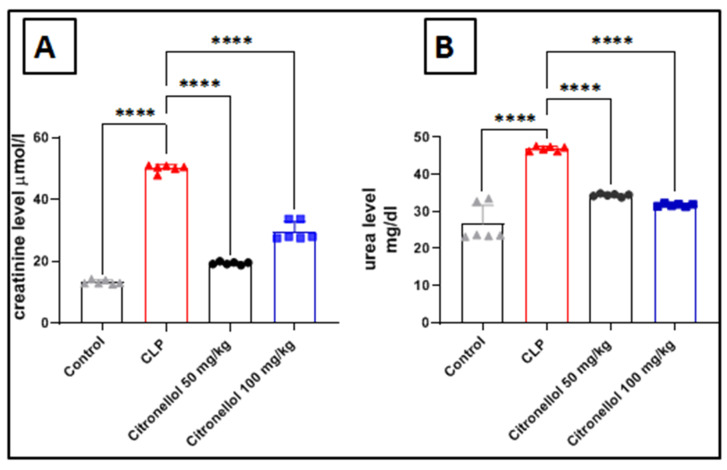
Effect of citronellol on urea and creatinine in CLP-induced kidney injury. Data represent mean ± SEM for urea and creatinine measured 12 h after induction with CLP in mouse kidney tissue: (**A**) creatinine; (**B**) urea. Citronellol treatment used two different doses (50 mg/kg and 100 mg/kg) for four consecutive days before CLP. *n* = 6 in each group. **** *p* < 0.0001 indicate statistical significance. Control: 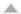; CLP: 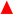; citronellol 50 mg/kg: 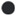; citronellol 100 mg/kg: 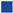.

Assessing urea and creatinine levels in mice with CLP-induced kidney injury provides an effective method for evaluating the effects of the injury and treatment on kidney function. Increased serum levels of urea and creatinine indicate compromised kidney functionality, which may arise from several factors, including injury caused by sepsis. Our studies show that CLP in mice leads to a decline in renal function, as reflected by a notable rise in serum urea and creatinine levels (urea: 46.92 ± 0.25 vs. 26.69 ± 2.032 mg/dL; creatinine: 50.11 ± 0.48 vs. 13.21 ± 0.27 µmol/L) when compared to control mice (Figure 4A,B). Additionally, mice that received treatment with citronellol at doses of 50 mg/kg or 100 mg/kg exhibited reduced increases in urea and creatinine, demonstrating improved renal function as evidenced by lower serum levels of these markers (urea: 34.37 ± 0.15 and 31.69 ± 0.16 vs. 46.92 ± 0.25 mg/dL; creatinine: 19.32 ± 0.19 and 29.70 ± 1.26 vs. 50.11 ± 0.48 µmol/L) compared to those observed in the CLP group.

### 3.4. Effect of Citronellol on KIM-1 in CLP-Induced Kidney Injury

**Figure 5:**
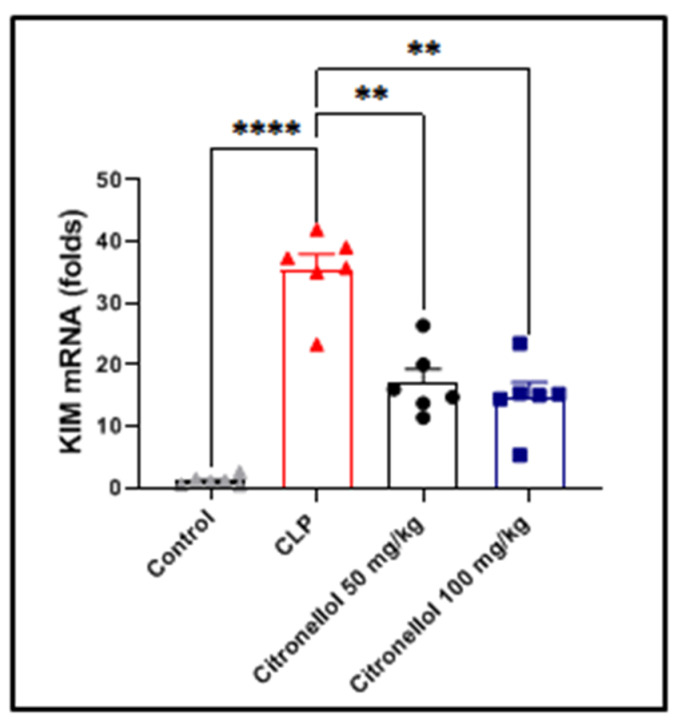
Effect of citronellol on KIM-1 in CLP-induced kidney injury. Data represent mean ± SEM for KIM-1 mRNA level of markers measured 12 h after induction with CLP in mouse kidney tissue. Citronellol treatment used two different doses (50 mg/kg or 100 mg/kg) for four consecutive days before CLP. Calculations of gene expression were performed relative to GAPDH level as a control gene in fold changes. *n* = 6 in each group. ** *p* < 0.01; **** *p* < 0.0001 indicate statistical significance. Control: 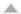; CLP: 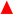; citronellol 50 mg/kg: 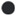; citronellol 100 mg/kg: 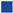.

To further examine the effect citronellol on tubular injury, KIM-1 mRNA expression was measured. In mice subjected to CLP, there was a notable increase in KIM-1 mRNA levels when compared to the corresponding levels in control mice (35.37 ± 2.63 vs. 1.19 ± 0.32 folds; Figure 5). Additionally, pretreating the mice with citronellol at both 50 mg/kg and 100 mg/kg doses led to a significant reduction in KIM-1 gene expression (17.04 ± 2.19 and 14.79 ± 2.35 vs. 68.37 ± 13.07) in comparison to the untreated CLP-induced mice. This finding suggests that citronellol provides a substantial protective effect against kidney injury caused by CLP.

### 3.5. Effect of Citronellol on Kidney Histology in CLP-Induced Kidney Injury

**Figure 6:**
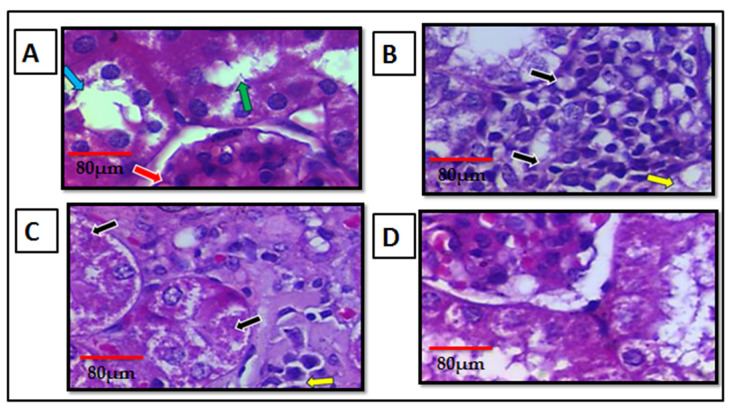
Effect of citronellol on kidney histology in CLP-induced kidney injury. Photomicrograph of cortex area of the kidney of negative control group mice (**A**), CLP group mice (**B**), 50 mg/kg citronellol-treated mice (**C**), 100 mg/kg citronellol-treated mice (**D**). H&E. (×400 magnification). Citronellol treatment used two different doses (50 mg/kg or 100 mg/kg) for four consecutive days before CLP. *n* = 6 in each group.

To further evaluate the effect of citronellol on the kidney dysfunction caused by CLP, a histopathological evaluation of kidney tissue was performed. The mouse kidneys were stained by hematoxylin and eosin (H&E). The pathological changes in the kidney tissue samples were examined by a specialized pathologist in a blinded manner under a light microscope at ×400 magnification and estimated using a semi-quantitative scoring system. Figure 6A shows normal histological architectures of cortex area of the kidney, no infiltration of inflammatory cells nor necrosis features. Note that the glomerulus is indicated by the red arrow, proximal convoluted renal tubules by the green arrow, and distal convoluted renal tubules by the blue arrow. Figure 6B shows the necrosis of the epithelial cells of renal tubules; these tubules formed spaces that filled with inflammatory cells (black arrow). However, some renal tubules showed necrosis features in the epithelial cells, where pyknosis, karyorrhexis, or karyolysis of epithelial cells nucleus were observed (yellow arrow). The pathological lesions were observed in most of the cortex area of the affected kidney. Figure 6C shows the mild infiltration of inflammatory cells (black arrow) near the glomerulus. However, some cortical renal tubules near the inflammatory cells showed necrosis features (yellow arrow). The pathological lesion was observed in less than 20% of the cortex area of the affected kidney. Figure 6D shows the normal histological architecture of the cortex area of the kidney; there was no infiltration of inflammatory cells or any necrosis features. Table 2 shows the results of AKI score.

## 4. Discussion

Acute kidney injury (AKI) occurs in about 13.3 million people per year [26,27]. It has been established that sepsis-induced AKI causes considerable damage mediated by the inflammatory response. AKI often occurs because of acute illnesses and is linked to significant rates of complications and fatalities during hospitalization. There is a growing interest in the long-term effects on individuals experiencing AKI. Evidence suggests a notably higher long-term risk of developing chronic kidney disease (CKD), progressing to end-stage kidney disease (ESKD), as well as an increased mortality rate following an AKI episode. These consequences are associated with high healthcare resource utilization, create a major financial burden for medical services, and impose considerable pressure on facilities that offer ongoing renal replacement therapy [28].

Research has shown that AKI caused by sepsis results in substantial injury driven by the inflammatory response. The cecal ligation and puncture (CLP) model induces sepsis due to stercoral peritonitis, which is followed by the translocation of multiple microbes into the bloodstream and an initial inflammatory response [23]. It is now understood that the inflammatory cascade, both macrovascular and microvascular dysfunction, along with abnormal cellular response, are the three fundamental components of the pathophysiological mechanisms underpinning sepsis-related AKI [2].

In this study, a model of AKI was established by CLP surgery in mice. It was found that CLP induced renal dysfunction by increasing serum urea and creatinine. In addition, CLP-induced AKI also causes renal pathological changes, including a tubular cell sloughing, the loss of the brush border, and tubular dilation in the cortex. Our results are consistent with those of previous studies [29,30].

The outcomes of our animal studies offer important insights into the possible health advantages of citronellol, particularly in terms of reducing the negative effects caused by CLP in mice. A significant finding of this research is that the mice displayed various signs of illness, such as malaise, fever, chills, piloerection, generalized weakness, decreased gross motor activity, increased clustering, and diminished appetite after undergoing CLP. These symptoms reflect the immune response activated by CLP, which may involve inflammation, fever, and a spectrum of behavioral and physiological alterations. What makes this investigation particularly intriguing is the noticeable difference in the symptoms of the mice following the administration of citronellol. The improvement in the health and well-being of the mice treated with citronellol suggests that it may act as a potential countermeasure against the detrimental effects of CLP. In our studies, the assessment of mouse well-being involved evaluating MSS and survival rates. Consistent with earlier studies, mice subjected to CLP displayed heightened MSS and increased mortality, pointing to a decline in their health status. This corresponds with findings from other investigations that have established that CLP application leads to a high MSS and heightened mortality rates in mice [31].

Upon treatment with citronellol (50 mg/kg or 100 mg/kg), there was a reduction in MSS and an enhancement in survival rates compared to the CLP group. This implies an improvement in the condition of mice experiencing kidney inflammation due to CLP, augmenting the results obtained from the assessment of other inflammatory markers. These findings are further substantiated by the measurements of urea and creatinine levels, which were elevated in the CLP group reflecting additional renal impairment, as also reported by other studies [29,30]. Moreover, administering citronellol resulted in lowered urea and creatinine levels, indicating a protective role against renal damage.

Our research also delivers a thorough perspective on the potential therapeutic advantages of citronellol in addressing the inflammatory and oxidative stress responses triggered by CLP in mice. Several crucial points emerge from our investigations. Citronellol treatment significantly diminished the production of inflammatory cytokines, such as TNF-α, compared to the mice subjected solely to CLP. These observations are consistent with prior research that has demonstrated the anti-inflammatory characteristics of citronellol in various animal models [32,33]. TNF-α is one of the cytokines responsible for the activation and recruitment of inflammatory cells, which ultimately causes the stimulation of vascular endothelium, nitric oxide release, and, accordingly, local vasodilation and increased vascular permeability [34,35]. The CLP surgery in group 2 (the CLP group) exhibited a marked increase in TNF-α levels, which is in line with results from earlier studies [36,37,38]. Conversely, citronellol treatment in groups 3 and 4 (citronellol 50 mg/kg + CLP and citronellol 100 mg/kg + CLP, respectively) led to decreased TNF-α production when compared to the mice that underwent CLP in group 2. Our results align with previous research, confirming the anti-inflammatory effects of citronellol in various animal models [39]. In addition, other studies have explored the anti-inflammatory effects of different terpenes across various experimental frameworks [40,41].

In our investigation, we evaluated the mRNA levels of NF-ĸB and AP-1 to assess the impact of citronellol on the MyD88-dependent signaling pathway [42]. We observed remarkable results regarding the mRNA expressions of NF-κB and AP-1; NF-κB and AP-1 were notably upregulated in the CLP group when contrasted with the control group. These findings are consistent with previous research that indicates that CLP in a mouse model triggers the gene expression of NF-κB [43].

Our findings also indicated that citronellol exhibited a notable anti-inflammatory impact, as daily pretreatment with 50 mg/kg and 100 mg/kg of citronellol for four days significantly reduced the gene expression of NF-ĸB and AP-1 in kidney tissues. Numerous studies have documented that citronellol was previously examined for its anti-inflammatory properties in an animal model by lowering NF-κB expression [42,44]. NF-ĸB has been recognized as a key mediator in the release of pro-inflammatory cytokines and plays a positive role in regulating the expression of various genes associated with the acute phase response, including TNF-α, IL-6, and other inducible enzymes.

Our study showed that CLP resulted in the elevated mRNA expression of AP-1; this was significant in the renal tissue when compared to that of the control group. Additionally, previously notable anti-inflammatory actions were observed by downregulating TNF-α generation in CLP-activated macrophages as a consequence of the inhibition of the AP-1 and NF-kB signaling models [45]. Citronellol showed similar effects, which could confirm the potential effect of terpene in CLP-induced AKI. Accordingly, the downregulation of citronellol to NF-κB and AP-1 expressions in the current study could clarify the substantial anti-inflammatory role of citronellol in targeting upstream events that resulted in NF-κB and AP-1 activation through TLR4/MyD88, which is dependent on CLP-induced AKI [46].

According to a previous study [47], and as shown in Figure 5, mice that had been exposed to CLP surgery to induce sepsis had considerably higher levels of KIM-1 than mice in the normal control group after CLP surgery. It is interesting to note that the KIM-1 gene expression was significantly decreased when citronellol (50 or 100 mg/kg) administered before CLP surgery was performed [17,32].

## 5. Conclusions

In summary, our results highlighted the importance of the natural bioactive molecules. Without doubt, our studies need to be revisited and explored for their pharmacological effects in various pathological conditions. Citronellol, as a natural active monoterpene, showed a remarkable protective effect in the kidney, which is suggested to be produced by its strong anti-inflammatory action. Citronellol effectively remedied sepsis-induced AKI, an effect that necessitates further research to explore its molecular effect and its toxicological profile in more detail. We remain hopeful that the therapeutic benefit of citronellol for human AKI will be explored in clinical settings.

## Figures and Tables

**Table 1:** Primer sequences.

Primers		Sequence 5 ʹ→ʹ3ʹ Direction	Accession No.
GAPDH	Forward	CGGGTTCCTATAAATACGGACTG	NM_001289726.2
GAPDH	Reverse	CCAATACGGCCAAATCCGTTC	
NF-ĸB	Forward	AAGACAAGGAGCAGGACATG	NM_001410442.1
NF-ĸB	Reverse	AGCAACATCTTCACATCCC	
AP-1	Forward	AG GCTGCAGGATGATGCGAT	NM_007457.4
AP-1	Reverse	TTCTAGCCAGGACGACTTGC	
KIM-1	Forward	GGCTCTCTCCTAACTGGTCA	XM_011248784.3
KIM-1	Reverse	CCACCACCCCCTTTACTTCC	
TNF-α	Forward	TAGCCCACGTCGTAGCAAAC	NM_013693.3
TNF-α	Reverse	ACAAGGTACAACCCATCGGC	

**Table 2:** AKI score.

Group	Mean ± SD
CLP	3.40 ± 0.55 *
Citronellol 50 mg/kg	1.60 ± 0.55 **
Citronellol 100 mg/kg	0.40 ± 0.12

*: indicate a significant (*p* < 0.05) difference between the CLP group compared with other groups. **: indicate a significant (*p* < 0.05) difference between the citronellol 50 mg/kg group compared with the citronellol 100 mg/kg group.

## Data Availability

The data presented in this study are available on request from the corresponding author.
